# Development of Vegetation-Pervious Concrete in Grid Beam System for Soil Slope Protection

**DOI:** 10.3390/ma10020096

**Published:** 2017-01-24

**Authors:** Xiaohua Bao, Wenyu Liao, Zhijun Dong, Shanyong Wang, Waiching Tang

**Affiliations:** 1 College of Civil Engineering, Shenzhen University, Shenzhen 518060, China; bxh@szu.edu.cn (X.B.); wlwt7@mst.edu (W.L.); 2 ARC Centre of Excellence for Geotechnical Science and Engineering, The University of Newcastle, Callaghan NSW 2308, Australia; Shanyong.Wang@newcastle.edu.au; 3 School of Architecture and Built Environment, The University of Newcastle, Callaghan NSW 2308, Australia; patrick.tang@newcastle.edu.au

**Keywords:** vegetation-pervious concrete, alkalinity reduction, admixture, strength, permeability, slope protection

## Abstract

One of the most efficient and environmentally friendly methods for preventing a landslide on a slope is to vegetate it. Vegetation-pervious concretes have a promising potential for soil protection. In this study, the vegetation-pervious concrete with low alkalinity was developed and studied. Combined with a grid beam structure system, the stability and strength between the vegetation-pervious concrete and base soil are believed to be enhanced effectively. For improving plant adaptability, the alkalinity of concrete can be decreased innovatively by adding a self-designed admixture into the cement paste. The effects of the admixture content on alkalinity and compressive strength of the hardened pervious concrete were investigated using X-ray diffraction (XRD) and compression test, respectively. Meanwhile, the permeability of the vegetation-pervious concrete was studied as well. Through comparing with ordinary pervious concrete, the effect of low alkaline pervious concrete on vegetation growth was investigated in a small-scale field for ten weeks. The test results indicated that the alkalinity of the cement samples decreased with the increase of admixture content, and the vegetation grew successfully on previous concrete. By increasing the admixture content to approximately 3.6%, the compressive strength of pervious concrete was more than 25 MPa.

## 1. Introduction

Cement based materials, such as concrete and cement mortar, are one of the most widely used building materials in world. With development of science and technology, concretes with special functions were prepared and developed [1,2]. More and more structural-functional concretes have been utilized in civil engineering.

Slopes are the most common landforms on Earth. Excluding natural slopes, large-scale infrastructure and urbanization constructions have created a large number of man-made slopes in China [3,4,5]. Unprotected (bare or uncovered) man-made slopes are at risk of different landslide types. United States geological survey (USGS) reports thousands of major landslides each year, many of which are triggered by rainfall [6]. Earthflow is one kind of landslide, and it is easily caused by rainfall in the case of unprotected slopes.

**Figure 1:**
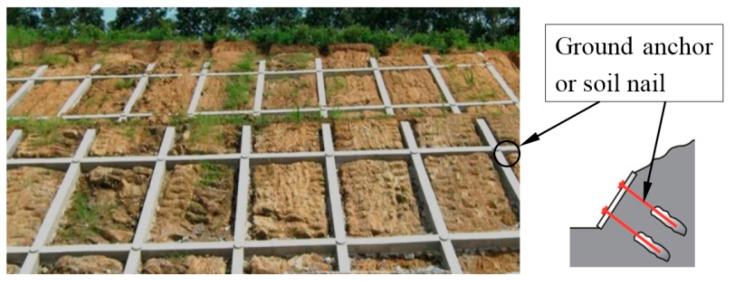
A typical application of grid beam structure on man-made slope.

Continuous grid beam structures made of concrete have been used for many years in China to stabilize the natural and man-made slopes. Generally, this slope stabilization structure is called “Grid Beam”. Figure 1 shows a typical application of continuous grid beam structure on a man-made slope. In order to meet the requirement of slope stabilization, the continuous grid beam structures are often used in combination with ground anchors or soil nails. Unstable slopes can be effectively stabilized by concrete grid beams when ground anchors or soil nails are installed at the intersection of beams (see Figure 1).

However, the soils within the frame of grid beams are vulnerable to be washed away during heavy rain. One of the fastest and most environmentally friendly methods for preventing or remedying a runoff on a slope is to vegetate it [7]. Many researchers have studied the effects of vegetation on runoff [8,9,10,11]. However, the surface runoff may still occur when excess water, such as storm water, melt water or water from other sources flows over the slope surface. Apart from rainfall characteristics such as intensity, duration and distribution, there are a number of site (or catchment) specific factors that have a direct bearing on the occurrence and volume of runoff. For example, the infiltration capacity is dependent on the porosity of a soil, which determines the water storage capacity and affects the resistance of water to flow into deeper layers. Porosity differs from one soil type to the others. The highest infiltration capacities are observed in loose, sandy soils, whereas heavy clay or loamy soils have significantly lower infiltration capacities. The vegetation has an effect on the infiltration capacity of the soil.

**Figure 2:**
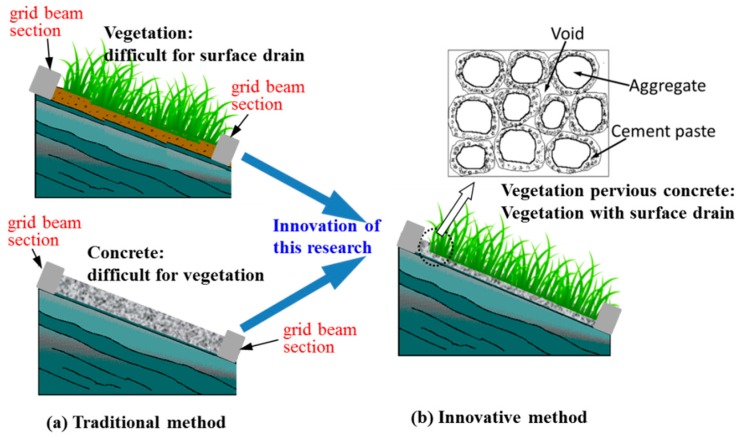
Function of vegetation-previous concrete with continuous grid beam structure frame for earthflow prevention.

In practice, surface drainage can successfully reduce the runoff of a slope [12]. Soil-cement cover is a common method for surface drainage. However, vegetation is difficult to grow on the surfaces that use this drainage method. Pervious concrete possesses a high porosity and very high strength compared with soil and a higher infiltration capacity compared with concrete [13,14]. Therefore, its potential for surface drainage is significant. If plants can grow on the surface of pervious concrete, that is, if vegetation-pervious concrete can be developed, surface vegetation and surface drainage can be combined for slope protection. Figure 2 presents the functions of vegetation-previous concrete in grid beam structure for preventing earthflow type landslide.

The ideal vegetation-pervious concrete consists of a large number of connected pores, which are filled with suitable materials that contain moisture and nutrients needed by plants. The plant roots are able to grow and extend through the pores to form an integrated system of concrete and plant matter. For application to the construction industry, several important characteristics should be considered: (1) vegetation-pervious concrete must feature high strength and relatively good durability [15,16], and a variety of plants must be able to grow on as in normal soil; (2) for retaining moisture and nutrients, it is essential to fill up the pores in concrete with vegetation matrix [17]; and (3) the vegetation-pervious concrete can either be prefabricated to effectively reduce the construction period or be cast onsite [18].

However, vegetation-pervious concrete, which is suitable for vegetation growth while maintaining water drainage and mechanical properties, is challenging to be obtained. The greatest challenge is the vegetal properties of this type of concrete. The hydration reaction of cement generates Ca(OH)_2_ that accounts for 20%–25% of hardened cement samples. Thus, the concrete is found to be strongly alkaline, with pH value up to 12–13 [19,20]. Due to its high alkalinity, hydrated Portland cement is not favourable to the growth of ground and aquatic plants in concrete. However, some greeneries can be planted on the slope to achieve greening effects. Even if some greeneries can grow on concrete surface, the effect of root growth on concrete structure and concrete strength needs to be considered. Pervious concretes have higher porosity [21] and high enough strength [22] for greeneries growth. For reducing the alkalinity of concrete, the research of [23] proposed the theories of morphological transformation of cement hydration, ion homeostasis for hydroxide ion (OH^−^) and molecular sieve effects based on their studies on the formation and manifestation of alkaline solution in concrete pores through a series of means such as physical, geotechnical and biochemical methods. They also proposed more than ten methods e.g., using a modifier to not only adjust the pH value in Portland cement concrete pores to 7–7.5, but also to increase their buffer capacity, which can be defined as the maximum amount of either strong acid or strong base that can be added before the occurrence of a significant change in pH. Consequently, the concrete was found more favourable to plant growth, and its durability was also improved.

Although conventional methods can reduce the alkalinity of pervious concrete, the main problem identified in current research of vegetation-pervious concrete is that the conventional methods of reducing alkalinity tend to weaken the physical and mechanical properties of vegetation-pervious concrete when compared to the control concrete. Therefore, the primary goal of this study was to propose a method to reduce the alkalinity of pervious concrete for vegetation at the same time maintaining the mechanical properties of the concrete. Chemical and mineral admixtures have been extensively used to adjust or modify the properties of fresh and hardened concrete [24]. In this research, the method of adding admixtures was employed to reduce the alkalinity of concrete. The objective of this research is to develop efficient and applicable vegetation-pervious concretes in gird beam structure for soil slope protection. A self-designed admixture was used to develop pervious concrete with low alkalinity that was suitable for vegetation growth. The properties of the vegetation pervious concrete with different admixture dosages were systematically investigated.

## 2. Materials and Methods

### 2.1. Materials and Mix Proportions

In this research, the method of adding a self-designed admixture, which is named Reducing Cement Alkaline (RCA), was employed to reduce the alkalinity of concrete. Table 1 lists the chemical components of the admixture RCA. It can be seen that silica fume is one the major components in RCA. Silica fume improved the compressive strength of porous concrete compared with normal concrete. Technically speaking, when the silica fume is added, more water is required for wetting the large specific surface area of silica fume particles in a concrete mixture to retain its workability. Thus, a polycarboxylate-type superplasticizer in RCA is necessary to prepare vegetation-pervious concrete. Therefore, the benefit of using silica fume was not achieved without other chemical admixtures.

Portland cement (PC) Type 42.5R, which conforms to British Standard BS EN 197-1 CEM I [25], was used to mix the vegetation-pervious concrete. Table 2 presents the chemical ingredients of the cement. The coarse aggregate is crushed limestone with a nominal grading size of 19 mm gravel. The pervious concrete was designed to have a continuous voids ratio of 27%. Table 3 shows the mix proportion of the vegetation-pervious concrete in this investigation.

In China, at present, the costs of the vegetation-pervious concretes produced by manufacturers are less than RMB 300/m^3^ (about USD 43/m^3^). Other researchers who are interested in similar area can do a cost benefit analysis based on the cost information and their project characteristics.

### 2.2. Sample Preparation

In this study, the procedures for sample preparation are described as follows. First, part of the mixing water was added into the coarse aggregate and stirred until the gravel surface was wet. Second, the cement was added and the mixture was stirred for approximately 30 s to allow the gravel surface being coated with a layer of cement paste. Third, the remaining mixing water and the admixture RCA were added. The mixture was then stirred continuously for about 3 min, until the cement paste coating on gravel thickened. Finally, the mixture was cast into a 100 mm × 100 mm × 100 mm mould in three steps. Moulds were filled with the mixed concrete up to one-third of the height of the mould and subjected to 25 times hand tamping. The same procedure was applied for the two-third and complete fill-ups. The vegetation-pervious concrete was removed from the mould after 24 h and cured for 28 days in a controlled environment (20 ± 2 °C, >98% RH), which is similar to the process for conventional concrete.

A control mix and four hardened cement paste mixes with different proportions of the admixture RCA (1.2, 2.4, 3.6 and 4.8 wt % of cement) were analysed. The water/cement ratio was 0.35. Cubes with a size of 100 mm were used for studying the compressive strength at 4, 7, and 15 days after standard curing in a controlled environment (20 ± 2 °C, >98% RH). The compressive test was performed according to the China national standard GB/T 50081-2002 [26]. Three specimens were used for each test and the mean compressive strength was reported. A new group of samples would be cast for repeated tests if the value of any sample exceeded ±15% range of average value.

### 2.3. Test Methods

In this study, different properties of vegetation-pervious concretes were investigated. Table 4 gives a summary of concrete sample size and numbers for each test in this study. All samples were used in the tests.

#### 2.3.1. Physical and Mechanical Properties

##### (a) Porosity

**Figure 3:**
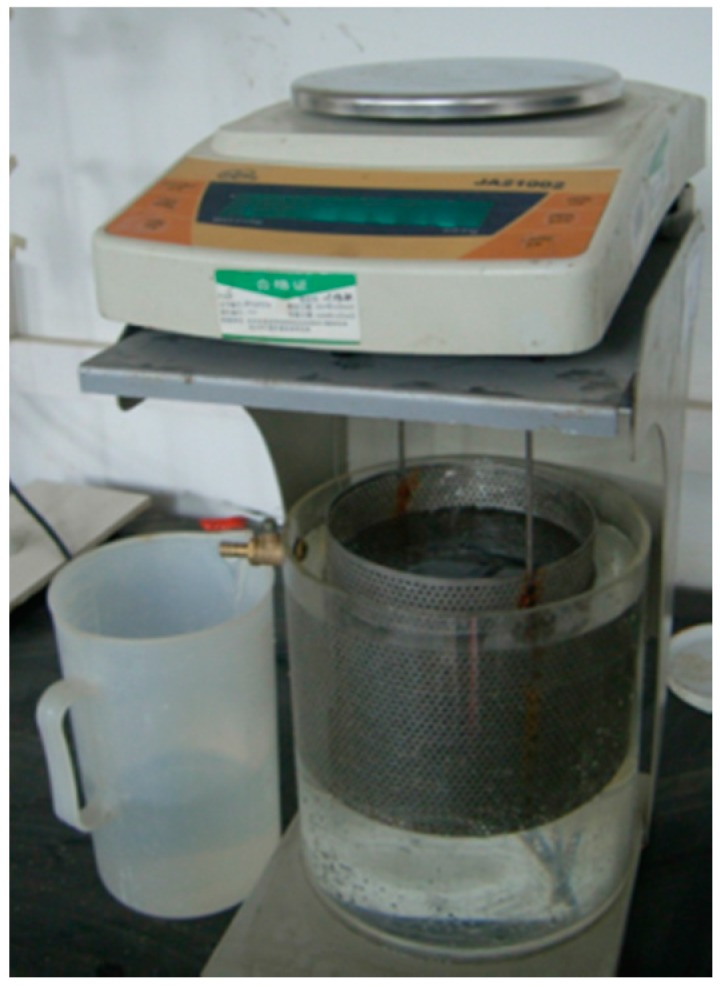
Hydrostatic balance employed in this study.

Sample used in this test is cylinder with the dimensions 100 mm (*D*) × 100 mm (*H*). The test procedure for the porosity of vegetation-pervious concrete was as follows:

(1) The sample was dried in an oven at 60 °C until the mass did not change, and the mass (*M*_1_) was measured.
(2) The diameter and height of the sample were measured, and the volume of the sample (*V*_cylinder_) was calculated.
(3) The samples will be soaked in water for 2 h for getting a saturated station. The mass of saturated sample (*M*_2_) was measured by a hydrostatic balance method as shown in Figure 3.
(4) The porosity (*P*) of the sample was calculated using the following equation:
  *P* = (*M*_1_ − *M*_2_)/(ϱ_water_ − *V*_cylinder_) × 100%,
  where *P* is the porosity of the sample, %; *M*_1_ is the sample mass in air, gram; *M*_2_ is the mass of the sample in water, gram, ϱ_water_ is the water density, g/mm^3^; and *V*_cylinder_ is the volume of the sample, mm^3^.

##### (b) Permeability

**Figure 4:**
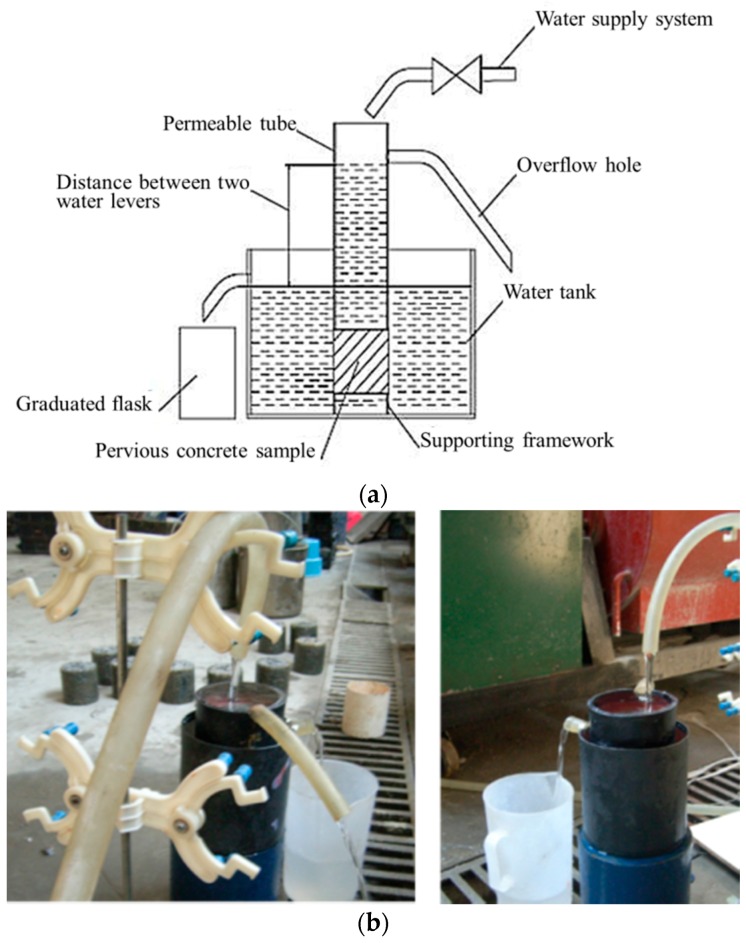
Device of permeable test for vegetation-pervious concrete. (**a**) A schematic drawing of the device; and (**b**) test process using the device.

The device that was employed for the permeability test for vegetation-pervious concrete is shown in Figure 4. This device is with reference to the permeability coefficient of the apparatus in China building materials industry standard JC/T945-2005 [27]. The test procedure is described as follows:

(1) The diameter (*D*) and thickness (*L*) of the cylindrical samples were measured twice using callipers, and the data were averaged to 0.1 cm. Then, the sample surface area (*A*) was calculated.
(2) The sidewall of the cylinder was sealed by a watertight material (epoxy). The epoxy was brushed on the sidewall of the samples evenly and the thickness of epoxy was more than 1 mm. The water was allowed to penetrate the sample from the upper and lower surfaces. After the watertight material was cured, the sample was placed in water to a water-saturated state before the test.
(3) The sample was not removed from the water until the permeability test was performed. The sample was connected with the permeable tube shown in Figure 4a and placed in a water tank; and the water supply valve was subsequently turned on. When the water overflowed from the overflow hole, the speed of the water supply was adjusted to maintain a certain level of permeability. After water stability was attained for the overflow hole and permeable tube, a graduated flask was used to collect water from the outlet of the water tank; the time *t* (seconds) and quantity of water outflow (*Q*) were measured three times, and the data were averaged.
(4) The distance (*H*), which was accurate to 0.1 cm, between the water level in the water tank and the water level of the permeable tube was measured by a rule.
(5) Permeability coefficient (*K*) of the sample can be calculated as the following equation:
  *K* = *Q* × *L*/(*A* × *H* × *t*),
  where *K* is the permeability coefficient of the tested sample, mm/s; *Q* is the quantity of water outflow within time *t*, mL; *L* is the thickness of the sample, mm; *A* is the upper surface of the sample, mm^2^; *H* is the distance between the levels, mm; and *t* is the testing time, s.

##### (c) Compressive Strength

Compressive strengths of the concretes were measured according to the method in China national standard GB/T 50081-2002 [26]. The final result is an average of three tested samples.

##### (d) Elastic Modulus

Because all sides of previous concrete cylinder were not smooth and it was impossible to test the samples directly without pre-processing the surfaces, both ends of previous concrete cylinders were required to cap before the modulus of elasticity test.

**Figure 5:**
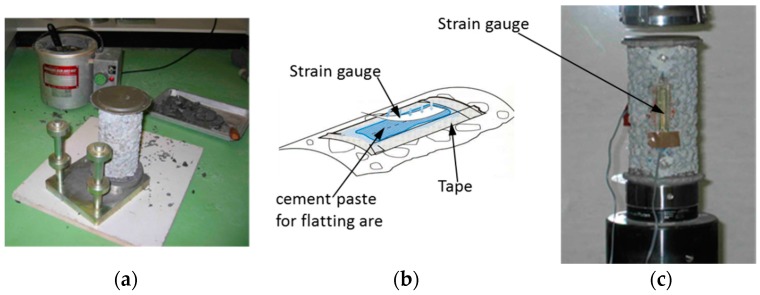
Testing process of elastic modulus of pervious concrete. (**a**) The equipment for capping cylinders and a capped cylinder; (**b**) a schematic drawing for how to attach strain gages; and (**c**) sample during testing.

The caps did not exceed a thickness of 10 mm at any point. Suitable capping materials may be a mixture of sulfur and fine siliceous sand or a mixture of sulfur and pulverized fuel ash [28,29,30]. In this research, the latter capping material was used. All sulfur type caps should be allowed to harden for at least two hours before the cylinder is tested. Figure 5a shows the capping equipment and a completed sample.

In this research, N2A series strain gages (Measurements Group Inc., Raleigh, NC, USA) were used to measure strains for calculating elastic modulus. Before sticking the strain gages, the two sides of the cylinders needed to be flattened using cement paste, and the sizes of flat areas were about 60 mm length and 20 mm width, and the position of the areas was the centre of the cylinder height. After the cement paste hardened, the strain gages were adhered at the flat areas. Figure 5b shows the method for adhering the strain gages and the sample that was tested. The testing method and calculating method were based on BS 1881: Part 121 [31] (method for determination of static modulus of elasticity in compression). Figure 5c shows a testing sample.

#### 2.3.2. Chemical Properties

After the compression test, the hardened cement pastes coated on the aggregate surfaces of the failure samples were collected and ground into powder to measure the amount of chemical elements in the hardened cement paste samples by using X-ray Diffraction (XRD). A Mercury AutoPore IV 9500 injection apparatus (Micromeritics, Norcross, GA, USA) was used for the pore analysis of the plain cement paste, and the XRD analysis was conducted using a DX—2500 X-ray diffractometer (Fangyuan, Dandong, China). These tests involved a Cu target, 3 kW of power, 0.03° per step and a scanning range from 5° to 70°. To avoid carbonation during grinding the cement pastes, the samples were ground in 100% alcohol and dried in a vacuum oven at 60 °C for 48 h.

#### 2.3.3. Vegetation Test in Laboratory

**Figure 6:**
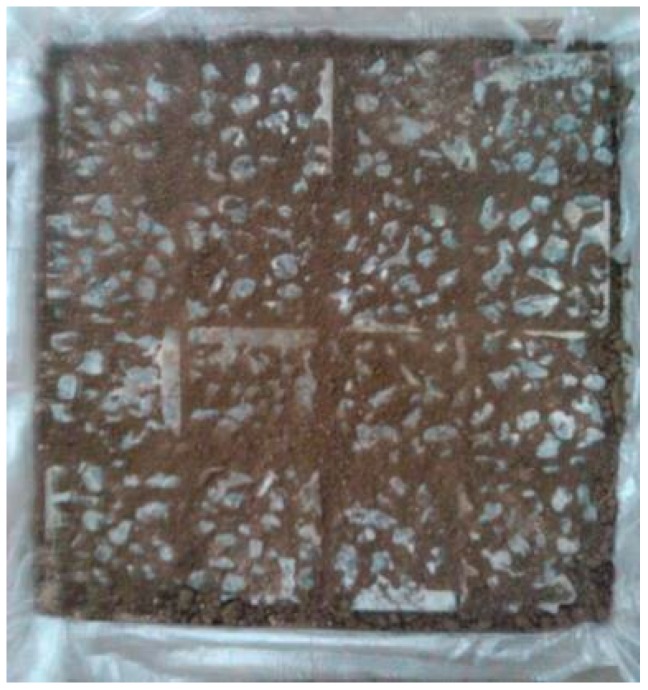
Vegetation-pervious concrete filled with mulching materials.

**Figure 7:**
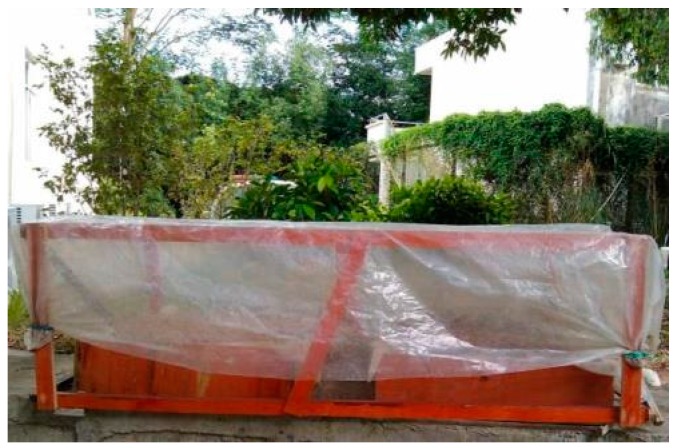
The vegetation-pervious concrete samples in a shed.

In this study, the vegetation-pervious concrete with 3.6% admixture RCA was chosen to conduct the vegetation test, and a pervious concrete without admixture RCA was used as a control sample. All concrete specimens were employed as vegetation beds and exposed to a natural environment. In the experiment, the vegetation-pervious concrete cubes were placed in a wooden box with the dimension of 400 (*L*) × 400 (*W*) × 150 mm (*H*). The pores of concrete specimens were grouted with mulching material (nutrient soil), which was able to retain water and fertilizer, as shown in Figure 6. The surface size of the pores is about 5–10 mm by rule measuring. Then, the concrete surface was covered with a 1–2 mm layer of nutrient soil, and the seeds of Bahia grass were sown on top of the concrete surface. The wooden boxes were placed outside in the early of April and the growth of Bahia grass was observed and recorded for ten weeks after sowing. The Bahia grass was watered two times (about 9:00 a.m. and about 6:00 p.m.) everyday. Based on the meteorological information from the Meteorological Bureau of Shenzhen Municipality [32], the range of temperature was about 16–25 °C and the range of relative humidity was about 65%–85%. Because heavy rainfall is usually observed in Shenzhen City during April to June, a shed with transparent plastic film was built to avoid heavy rain in the early growth stage. Figure 7 shows the shed used to protect the plants in the early growth stage.

## 3. Results and Discussion

### 3.1. Porosity, Permeability and Mechanical Properties

Table 5 shows a summary of the porosity, permeability and mechanical properties of the vegetation-pervious concrete. An increase in the admixture dosage caused a slight decrease in the porosity of the pervious concrete. A possible reason for this phenomenon is that adding the admixture can marginally increase the volume of cement paste, which can reduce the porosity of the resulting concrete. The porosity of the 28-day sample of each group is very similar and ranged from 22.1% to 23.4%.

The permeability coefficients of vegetation-pervious concretes, which are very similar, are approximately 1.8 cm/s. The similar porosity leads to similar permeability coefficient. Table 5 shows the relationship between the water permeability coefficient and porosity at various admixture dosages. The permeability coefficient tended to slightly decrease as the admixture dosage increased. Other studies on the permeability of pervious concrete [33,34,35] have shown that the water permeability coefficient has a large range for different concretes. Compared with the test results in the literature, for a similar strength level, the permeability coefficients observed in this research are twice those seen in other studies.

Compared with the effect of admixture on the porosity and permeability, the effect of admixture on the mechanical properties is significant. The compressive strength of the concrete increased considerably with the increase of admixture dosage. The highest increase is more than 60% (from 15.7 to 25.2 MPa). The higher strength and larger strength range of vegetation-pervious concrete yield an extensive range of applications for slope protection. Specifically, when the admixture content was 3.6%, the strength substantially increased to 25.2 MPa, which is much greater than the strength range of 5–15 MPa, as reported in the previous studies [16,36,37,38,39]. The main reason is that the admixture used can promote the cement hydration reaction. The 3-day to 28-day strength trend indicates that the admixture has a positive effect on the strength of the concrete samples in the early stage. For each group, the 14-day compressive strength is similar to the 28-day strength.

Elastic modulus and compressive strength are the two important properties used for the design of pervious concrete for slope protection. Although the effect of aggregates on compressive strength has been analysed, few studies have revealed the effect of admixture on the static modulus of elasticity of vegetation-pervious concrete. Static modulus can influence the deformation of vegetation-pervious concrete directly. As shown in Table 5, the effect of admixture on elastic modulus of vegetation-pervious concrete is less significant when compared to that of compressive strength. When the compressive strength vegetation-pervious concrete increased more than 60%, its elastic modulus only increased approximately 12.9% (from 20.1 to 22.7 GPa). Geotechnical engineers or researchers shall pay attention to the mechanical properties of the vegetation-pervious concrete when numerical simulation of slope stability is to be carried out.

### 3.2. Alkalinity of the Concrete

**Figure 8:**
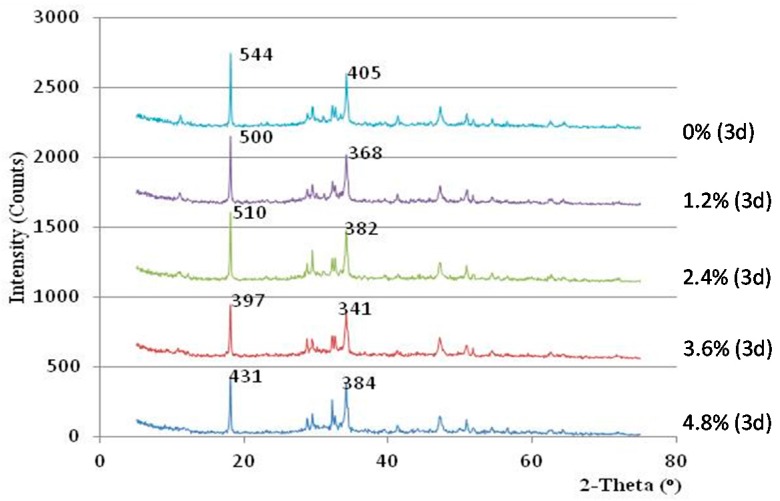
X-ray diffraction (XRD) curve of the hardened cement samples after three days of curing.

**Figure 9:**
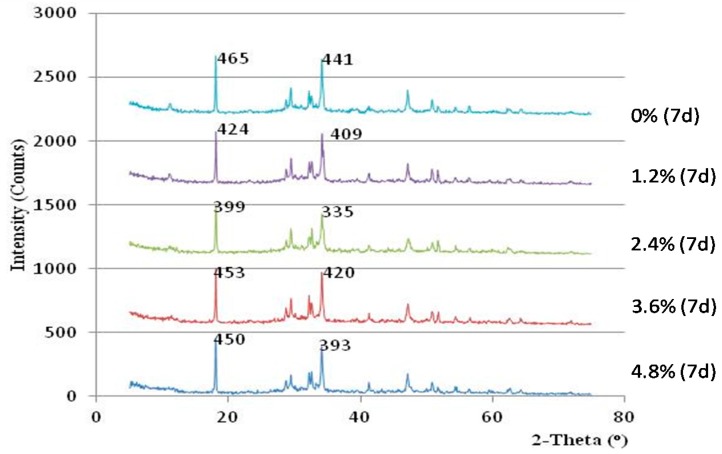
XRD curve of the hardened cement samples after seven days of curing.

**Figure 10:**
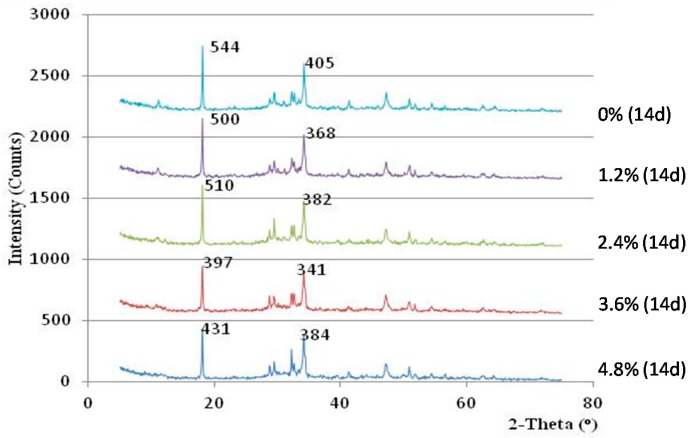
XRD curve of the hardened cement samples after 14 days of curing.

In this study, the alkalinities of hardened cement pastes from concretes with different admixture dosages were measured by XRD analysis. The levels of alkalinity were compared based on the amount of calcium hydroxide (Ca(OH)_2_) in the hardened cement samples. The intensity of the Ca(OH)_2_ crystal peaks in the XRD spectra was employed as an index to characterize the alkalinity of the sample. Figure 8, Figure 9 and Figure 10 display the XRD curves, which indicate the contents of Ca(OH)_2_ in the mixes for various curing times. The peaks in the XRD curves correspond to the mineral Ca(OH)_2_, and the intensity of the main peaks reflect the Ca(OH)_2_ contents in the cement samples.

The alkalinity of all cement paste mixes with admixture was initially lower than that of the cement paste mixes without admixture. The cement paste with admixture contents between 1.2% and 3.6% showed a decreasing trend in alkalinity with days of curing, and their alkalinity were even much lower than that of control samples after curing for 14 days (see Figure 10). However, the alkalinity of the cement sample with 4.8% admixture was found to be always higher than the control sample for the entire testing period. Thus, the dosage of the admixture content should be monitored between 1.2% and 3.6% to maximize the alkalinity reduction effect.

Based on the above results and observation, it can be known that when the admixture content between 2.4% to 3.6% is added, the strength of vegetation-pervious concrete can be improved, whereas its alkalinity can be reduced.

### 3.3. Comparison of Vegetation

**Figure 11:**
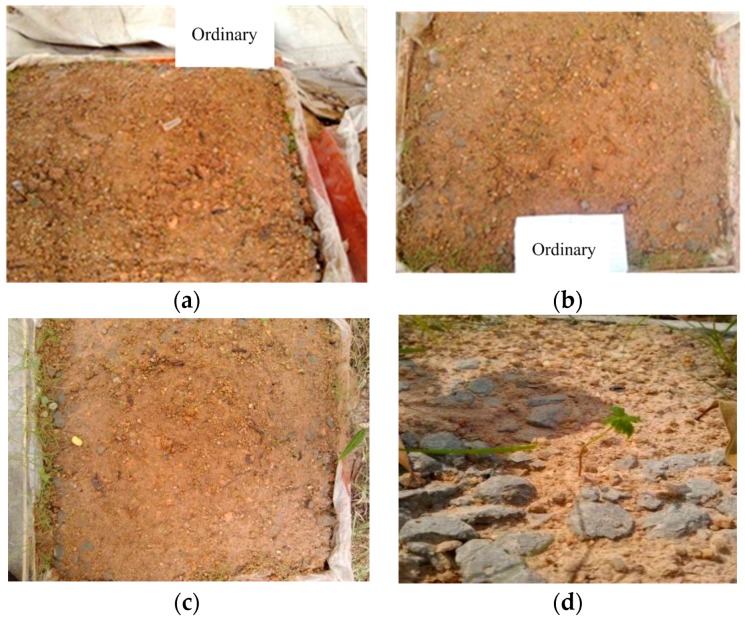
Vegetation growth after seeding on pervious concrete without vegetation admixture. (**a**) one week; (**b**) three weeks; (**c**) five weeks; and (**d**) 10 weeks.

The growing status of Bahia grass planted on the surface of ordinary vegetation-pervious concrete (without admixture) over ten weeks of exposure to the natural environment is shown in Figure 11.

As shown in these photos, Bahia grass started germinating typically one week after sowing; however, the grass began to wither after three weeks and died after five weeks. It is interesting to note that some Bahia grass and weeds were found to grow well in the surrounding soils or within the gap of concrete blocks in five weeks. After the ten-week observation, the plants eventually failed to grow on the concrete surface. Neither the soil in the pores of the concrete nor the soil above the concrete surface was suitable for plants because the alkalinity of this soil was higher than the alkalinity of the surrounding soil. This conclusion can be supported by XRD test results in Section 3.2 as well.

**Figure 12:**
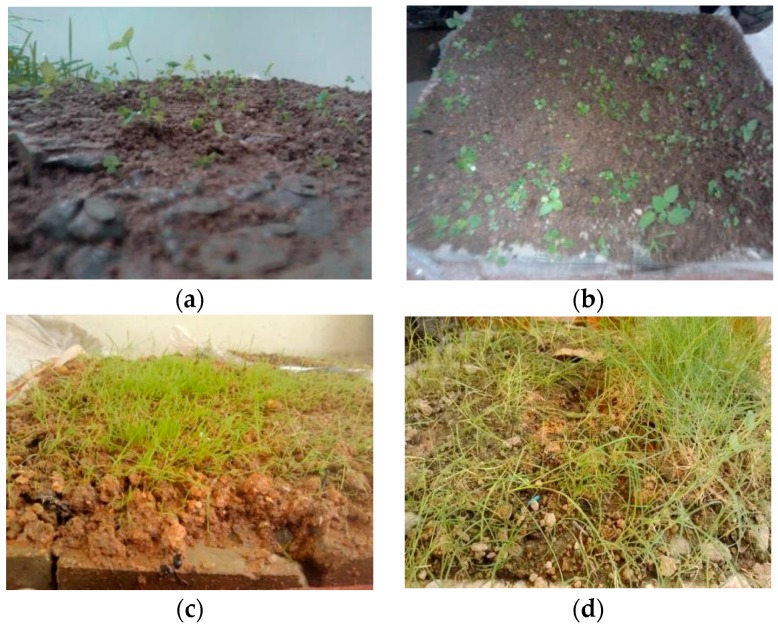

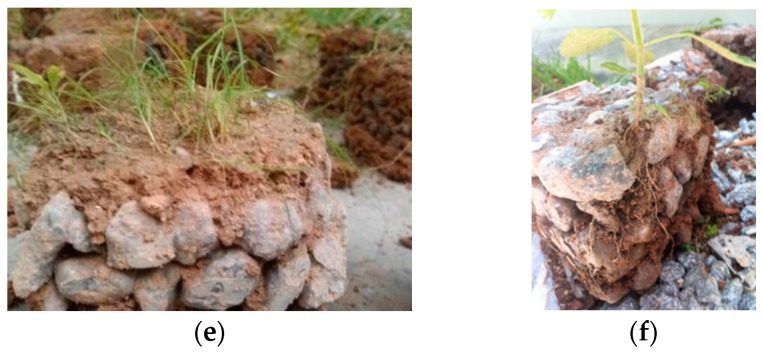
Vegetation growth after seeding on vegetative pervious concrete with 3.6% admixture after (**a**) one week; (**b**) three weeks; (**c**) five weeks; and (**d**) 10 weeks; (**e**) close-up view of the grass after ten weeks; and (**f**) roots grew through internal pores of vegetative pervious concrete.

Figure 12 presents the growing status of Bahia grass planted on the surface of vegetation-pervious concrete with 3.6% admixture at different stages.

As shown in Figure 12, Bahia grass planted on the upper surface of the vegetation-pervious concrete began growing at a faster rate in the first three weeks after sowing and continued to grow throughout the 10-week observation period. This indicates that the admixture RCA can successfully reduce the effect of cement paste on the alkalinity of the soil that fills the internal pores of the vegetation-pervious concrete. The silica fume (SiO_2_) among RCA additive chemically reacted with an alkaline substance (Ca(OH)_2_) to generate calcium silicate hydrates (C–H–S), which resulted in the decrease of alkalinity of the vegetation-pervious concrete and formation of a dense layer that effectively prevented the alkaline substance dissolving out from the concrete to the soil.

## 4. Conclusions

This paper demonstrated the use of an admixture method to reduce the alkalinity of vegetation-pervious concrete to create favourable conditions for vegetation growth. A vegetation-pervious concrete for soil slope protection was developed. Based on the results presented here, the following conclusions were drawn:

(1) The compressive strength and elastic modulus of the vegetation-pervious concretes increased with the increase of admixture, but the effect of admixture on elastic modulus was less significant compared to the compressive strength. The result showed that vegetation-pervious concretes with admixture showed lower alkalinity and higher strength. Therefore, the admixture used in this study was not only able to decrease the alkalinity of cement effectively but also improve the strength to as high as 25.2 MPa at an admixture content of 3.6%.
(2) In comparison, the alkalinity of the cement cube samples with admixture was lower than that of the cement paste mixes without admixture. When the admixture content between 2.4% to 3.6% was added, the strength of vegetation-pervious concrete was found improved, whereas its alkalinity was reduced.
(3) Compared with a traditional method, the developed vegetation-pervious concrete slope protection method can be adapted for different vegetation requirements and slope gradients by adjusting alkalinity and porosity of the concrete.
(4) Based on the test results of alkalinity, permeability coefficient and mechanical properties of the studied concretes, the vegetation-pervious concretes have demonstrated their potential suitability when using in continuous grid beam structure for slope protection.

## Figures and Tables

**Table 1:** The chemical compositions and functions of the admixture Reducing Cement Alkaline (RCA).

Ingredient	Ratio (wt %)	Function
Silica fume (SiO_2_)	37.8	To react with Ca(OH)_2_ and producing more calcium silicate hydrate (C–S–H) gel in cement paste to reduce alkalinity and to fill the pores in cement paste.
Ultra-fine calcium carbonate (CaCO_3_)	7.6	To fill the fine pores in cement paste with an inert material so that the cement paste becomes denser and reduces the Ca(OH)_2_ dissolution.
Polycarboxylate-type superplasticizer	7.5	To reduce water usage of cement paste and improve strength, to reduce the consumption of cement and reduce alkalinity.
Sodium Nitrite (NaNO_2_)	2.3	To improve the viscosity of the cement paste to enable the cement paste to effectively attach to the aggregate surface and improve the early strength of cement paste.
Polyacrylamide (–CH_2_CHCONH_2_–)	0.4	A water retainer to improve the viscosity of the cement paste.
Water	44.4	As the solvent in the admixture

**Table 2:** Chemical composition expressed as oxides (wt %) of cement.

Oxides (wt %)	CaO	SiO_2_	Al_2_O_3_	Fe_2_O_3_	K_2_O	MgO	TiO_2_	Si/Ca
OPC	64.6	21.10	5.90	3.10	-	1.00	-	0.327

**Table 3:** Mix proportions of vegetation-pervious concrete (1 m^3^).

Group Number	Ratio of Water to Cement	Admixture RCA Dosage (wt % of Cement)	Mix Proportion				
			Water Content in the Admixture (kg)	Water (kg)	Cement (kg)	Coarse Aggregate (kg)	Admixture RCA (kg)
VPC-0	0.35	0%	0	105	300	1600	0
VPC-12	0.35	1.2%	1.6	103.4	300	1600	3.6
VPC-24	0.35	2.4%,	3.2	101.8	300	1600	7.2
VPC-36	0.35	3.6%	4.8	100.2	300	1600	10.8
VPC-48	0.35	4.8%	6.4	98.6	300	1600	14.4

VPC-XX, VPC denotes vegetation-pervious concrete; XX indicates dosage of the admixture.

**Table 4:** Summary of concrete samples for the tests.

Categories	Sample Type and Size	Sample Number/Group
Porosity	Cylinder, 100 mm (*D*) × 100 mm (*H*)	3 samples
Compressive strength	Cube, 100 mm × 100 mm × 100 mm	3 samples
Elastic modulus	Cylinder, 100 mm (*D*) × 200 mm (*H*)	3 samples
Permeability	Cylinder, 100 mm (*D*) × 50 mm (*H*)	3 samples

*D* and *H* means diameter and height, respectively.

**Table 5:** Summary of porosity, permeability and mechanical properties of the concretes.

Group Number	28 Days Porosity *P* (%)		28 Days Permeability Coefficient *K* (mm/s)		Compressive Strength (MPa)								Elastic Modulus (GPa)			
					3 Days		7 Days		14 Days		28 Days		14 Days		28 Days	
	Mean	SD	Mean	SD	Mean	SD	Mean	SD	Mean	SD	Mean	SD	Mean	SD	Mean	SD
VPC-0	23.4	1.91	18.7	0.67	10.7	0.37	13.1	1.10	14.8	0.68	15.7	0.98	19.7	0.67	20.1	0.81
VPC-12	23.1	1.10	18.5	0.59	16.6	0.51	19.0	0.87	20.9	1.16	21.3	1.21	21.1	0.90	21.6	0.34
VPC-24	23.1	1.84	18.1	0.56	19.0	0.43	20.9	0.41	21.2	0.94	22.4	0.79	20.9	1.06	21.8	0.92
VPC-36	22.9	0.96	18.3	0.70	21.6	0.80	23.1	0.62	23.9	1.11	25.2	0.91	21.5	0.89	22.3	0.89
VPC-48	22.1	1.39	17.9	0.59	20.0	0.69	22.0	0.81	23.3	0.31	24.8	0.68	22.1	0.94	22.7	1.03

SD means standard deviation.
